# Greening Ultrasound-Assisted Extraction for Sorghum Flour Multielemental Determination by Microwave-Induced Plasma Optical Emission Spectrometry

**DOI:** 10.1155/2021/9201094

**Published:** 2021-12-06

**Authors:** María Isabel Curti, Florencia Cora Jofre, Silvana M. Azcarate, José M. Camiña, Pablo D. Ribotta, Marianela Savio

**Affiliations:** ^1^Facultad de Ciencias Exactas y Naturales, Universidad Nacional de La Pampa, Av. Uruguay 151, Santa Rosa L6300XAI, La Pampa, Argentina; ^2^Instituto de Ciencias de La Tierra y Ambientales de La Pampa (INCITAP), Mendoza 109, Santa Rosa L6302EPA, La Pampa, Argentina; ^3^CONICET-UNC. Instituto de Ciencia y Tecnología de Alimentos Córdoba (ICYTAC), Córdoba, Argentina; ^4^Universidad Nacional de Córdoba, Facultad de Ciencias Exactas,Físicas y Naturales, Córdoba, Argentina

## Abstract

Sorghum is the fourth most important cereal produced in Argentina and the fifth worldwide. It has good agronomic characteristics and could be developed in arid areas, allowing a wide geographic distribution. Its starch content, higher than 70%, makes it possible to obtain a good yield of flours. Nutritionally, it should be noted that the grain does not have the protein fraction called prolamins, which makes it suitable for consumption by people with celiac disease. The multielemental composition constitutes an important indicator of the nutritional profile of the grains and allows, together with other parameters, to select the most suitable varieties for human consumption. In its determination, the preanalytical stage is decisive to obtain a reliable result. Organic samples are a challenge for sample introduction systems that use plasma-based techniques. As an alternative to conventional pretreatment with a microwave-assisted digestion (MWAD), a greener, quick, and simple treatment is proposed, using ultrasound-assisted extraction (UAE) in diluted acid media. The UAE method accelerates analysis times, improves performance and productivity, and was applied to sorghum samples cultivated in the province of La Pampa (Argentina). Microwave-induced plasma optical emission spectrometry (MIP OES) was employed for the determination of Cu, K, Mg, Mn, P, and Zn. The detection limits found ranged from 0.6 (Cu) to 89 (P) mg kg^−1^, and the precision expressed by the relative standard deviation (RSD) was ≤7.7% (Zn). For validation, a maize reference material (NCS ZC 73010) was evaluated. The principal component analysis revealed three different groupings related to the sorghum varieties' mineral profile.

## 1. Introduction

Sorghum (*Sorghum bicolor* L. Moench) is the fifth cereal produced worldwide [1]. Its relative plasticity at planting time permits the development in arid areas [2, 3]. Sorghum grains are principally utilized for animal feeding and exportation in agricultural countries. However, it is an important source of nutrients especially for inhabitants of Asia and Africa who consume it in simple preparation [3]. In this regard, sorghum plays an important role in the nutrition of the most food-insecure regions of the world.

In the last few years, sorghum grain has increased recognition for its health-promoting benefits. The pericarp is rich in phenolic compounds, micronutrients, and dietary fiber, while in the endosperm starch is the main component [4, 5]. From a mineral nutrition point of view, the grains contain minerals such as phosphorus, magnesium, and potassium in varying amounts depending on the hybrids and are also an important source of iron and zinc, comparable to rice and wheat [6]. Additionally, a highlight attribute is the lack of gluten, which makes it suitable for consumption by people with celiac disease [7, 8].

The mineral content of sorghum flours is nutritionally relevant as an affordable source of nutrients. Sorghum is utilized in a wide range of food products such as beverages, porridges, and flatbreads [9]. The use of sorghum flour is also appreciated for being a gluten-free raw material available for baked products production. Numerous studies endorse the use of sorghum in the elaboration of bread, cakes, and cookies with good technological and sensory properties [10–14].

Green and equitable analytical chemistry focus on environmentally friendly analytical procedures application, highlighting their availability to society [15]. Thus, much emphasis should be placed on making determinations with easily available instruments and nonpolluting procedures. Microwave-induced plasma optical emission spectrometry (MIP OES) stands out as a low-cost acquired, sensitive, robust, and selective technique; its versatility allows a complete mineral profile quantification [16–18].

For plasma-based techniques, such as MIP OES, a total sample matrix decomposition is needed to solubilize the analyte, but this is an analytical process stage in which many mistakes can be made [19]. The application of analytical sample preparation techniques is hampered by the significant cost of instruments, leading to the use of low-cost extraction units, known for domestic and everyday use. Several works determined sorghum mineral composition, reducing the organic matter to ashes and then restituting it in acid before analysis [20, 21]. However, microwave-assisted digestion (MWAD) is considered the state of the art in sample preparation as it reduces solvent consumption and is faster than acid digestion, combining high temperature and high pressure [22]. Notwithstanding, due to its low cost and for the unique conditions provided by acoustic cavitation, ultrasound-assisted extraction (UAE) has evolved in the last decades as an efficient alternative to MWAD, enhancing solid sample pretreatment, promoting green aspects such as energy requirements, and minimizing solvent consumption [23–26].

Hence, a simpler and greener sample preparation method for sorghum flours based on UAE is proposed, improving performance and productivity. In order to assess nutritional profile, the multielemental determination of different sorghum cultivars from La Pampa, Argentina, was performed by MIP OES. Moreover, principal component analysis (PCA) was performed to find similarities and differences among sorghum hybrids.

## 2. Experimental

### 2.1. Samples

Sorghum flours were obtained from commercial hybrids of brown sorghum grains provided by Instituto Nacional de Tecnología Agropecuaria (Estación Experimental Anguil, La Pampa, Argentina): TOB 41 T, TOB EXP 903, ACA 548, ACA XP GR 209, ACA GR 233, ARGENSOR 110 T, GEN 11T, GEN 311 T, SUMMER II, SPRING T 60, EXP 483, TAMAR 2 GR, HS 26 LT, PS (graniferous sorghums); ARGENSOR 151 DP, GEN 417 SLT 55 (dual-purpose sorghums); and GS1, GS2, GSD 3, GSD 4 (forage sorghums).

Sorghum hybrids used in this study were carefully cleaned and freed from foreign materials. Cleaned sorghum samples (approximately 500 g) were conditioned at 12% humidity before milling. After that, the grains were subjected to a high-speed action mill (FOSS Cyclotec™ 1093, Spain) with a 0.5 mm screen to produce the flour.

The sorghum flour samples were then packaged in a polyethylene bag and kept refrigerated (4-5°C). The samples were dried in an oven at 60°C for 2 h until analysis.

### 2.2. Instrumentation

Multielemental determination was executed by MIP OES Agilent MP 4100 (Santa Clara, USA) which encompasses an inert OneNeb nebulizer, a double-pass glass cyclonic spray chamber, and an SPS3 autosampler system. MIP OES owns a Czerny–Turner monochromator with a VistaChip charge-coupled device (CCD) array detector. MIP OES 4100 runs with an online N_2_ generator. For each analyte studied, the operating parameters and instrumental conditions such as nebulizer gas pressure (NGP) and viewing position (VP) were automatically optimized by the MP Expert software (Agilent Technologies), and they are shown in Table 1. An Anton Paar MW 3000 microwave system (Graz, Austria) and an Ultrasound (Testlab) with a power of 160W and frequency of 40 kHz were used for sample preparation (25 samples at time).

### 2.3. Reagents and Solutions

Ultrapure deionized water (resistivity of 18.2 mΩ) was produced by a Millipore ultrapurifier (Darmstadt, Germany). All reagents were of analytical grade and all solutions were ready using distilled-deionized water. A Berghof® subboiling distiller system (Eningen, Germany) was used to obtain concentrated HNO_3_ (Merck, Darmstadt, Germany). Hydrogen peroxide (30% (w/w), Sigma-Aldrich) was also employed.

For MIP OES analytical calibration, standard solutions for Cu, K, Mg, Mn, P, and Zn were prepared in a matrix of 0.14 mol L^−1^ HNO_3_, with a multielemental standard SCIENCE Plasma CAL.

All material used throughout the study was cleaned with 10% HNO_3_ for 24 h and rinsed several times with ultrapure water.

### 2.4. Ultrasound-Assisted Extraction

The UAE was carried out by accurately weighing masses around 0.5000 g of dry samples, and they were transferred to polypropylene metal-free flasks with a screw cap. A volume of 5.00 mL HNO_3_, 7 mol L^−1^, and 1.00 mL of H_2_O_2_ was added; then they were placed into a thermostated water bath at 100 °C for 30 min; after that, UAE was performed for 30 min. The obtained suspensions were diluted up to 30.00 mL. Digestions were made in triplicate. Also, blank reagents were run.

### 2.5. Microwave-Assisted Digestion

For comparative purposes, the MWAD was performed by accurately weighing masses around 0.5000 g of dry samples; they were transferred to the hermetically sealed 10 0 mL PTFE tubes. A volume of 5.00 mL HNO_3_, 7 mol L^−1^, and 1.00 mL of H_2_O_2_ was added. The tubes were located in the Anton Paar microwave system and the next microwave-assisted heating program was implemented: 1st step—15 min for ramp time up to 190°C and up to 1200 W; 2nd step—15 min for hold time at 190 °C, up to 1200 W. Afterward, samples were relocated in a volumetric tube and diluted up to 30.00 mL. Digestions were made in triplicate. Blank reagents were run.

### 2.6. Accuracy Evaluation

For method accuracy evaluation, experiments were performed using a certified reference material (CRM): Maize Flour (NCS ZC 73010 Mealie) from National Analysis Center For Iron and Steel (NCS) Testing Technology Co., Ltd. Reference materials were prepared using the same conditions proposed for the sorghum flour samples.

### 2.7. Data Analysis

#### 2.7.1. Calibration and Validation Sets

Calibration models for each element were performed using five concentration levels in triplicate, and a blank solution was always considered. All experiments were carried out three times, and data were expressed as mean range values.

The proposed method was validated in terms of the following performance parameters: linearity, limits of detection and quantification, and repeatability, achieved under the optimal working conditions. Limits of detection (LOD) and quantification (LOQ) were calculated based on the International Union of Pure and Applied Chemistry (IUPAC) recommendations. Repeatability and recoveries (95% confidence level) were calculated based on analyzed certified reference materials.

#### 2.7.2. Principal Component Analysis (PCA)

In order to understand the correlations between the calculated multiple mineral concentration and to consistently evaluate the sorghum genotypes, a PCA was conducted. PCA is useful to elucidate the complex nature of multivariate relationships and to understand the structure of multivariate complex datasets revealing hidden patterns [27]. Thus, PCA provides an overview of the main information contained in two-way data, which can be visualized in a space of two or three dimensions.

First, PCA models as exploratory tools to ascertain the most appropriate data pretreatment procedure and to find outliers and main patterns. Similarities and differences between samples were studied by observing the scores of the principal components, and the importance of variables was studied by the loadings plots. Accordingly, PCA was performed on a dataset arranged in a matrix (40 × 6).

#### 2.7.3. Software

Using MATLAB (2014b) (The Mathworks, Natick, MA, USA), calculations associated with calibrations, merit figures, recovery studies, and statistical tests, in addition to preprocessing data, correlation coefficients, and PCA, were performed. Environment-based routines (available in https://www.iquironicet.gov.ar/descargas/univar.rar) along with other home-based routines were employed [28]. Fisher's variance comparison was applied to evaluate the data homogeneity and heterogeneity. Calibrations were performed by adjusting the lines with the least-squares criterion and the linear interval was evaluated through an *F*-test. Significant differences between treatments were carried out through average comparison by *t*-test. All tests were evaluated at 95% confidence level.

## 3. Results and Discussion

### 3.1. Instrumental Optimization

In plasma-based atomic emission spectrometry techniques, such as MIP OES, efficient transport of particles through the sample introduction system and identical decomposition, as well as atomization/excitation processes of standards and samples, is desirable [29, 30]. Through the NGP, the sample solution turns into a finely divided droplet forming a fine mist, being transported into the atomization region. To enhance matrix handling, in MIP OES, the plasma torch is vertically oriented into the cavity, to avoid distortions in the plasma and unfavorable phenomena occurring with the sample transport; nevertheless, it has axial viewing for the finest detection limits and optimal sensitivity. In this instrumental arrangement, emission signals from the plasma are directed into the preoptic system, which works in conjunction with a fast-scanning monochromator (Czerny–Turner monochromator—600 mm focal length) and a charge-coupled device (CCD) detector [29].

Some of MIP OES' main limitations include some fixed parameters, such as a microwave-applied power fixed at 1000 W [29]. However, the equipment software carried out a scan of NGP and viewing position (VP), selecting the one with the highest intensity. Thus, to attain the best analytical performance, for each analyte wavelength, the VP was automatically optimized by the MIP OES. Also, the NGP was optimized to acquire the highest analytical signal for each element. For its relevant importance from a nutritional point of view, Cu, K, Mg, Mn, P, and Zn were selected as the majority minerals present in sorghum grains. Table 1 resumes the optimized parameters for each element.

### 3.2. Method Comparison: UAE vs. MWAD

In the analytical process, the sample preparation stage is decisive to obtain reliable results. Organic sample preparation is a challenge, and in the case of sorghum grains, the starch, protein, and fiber content may undergo extensive mineralization, before introduction to plasma-based techniques. In the last decades, UAE for trace analysis has dramatically expanded, reaching satisfactory outcomes leading to fast, efficient, and clean analytical sample preparation methods, achieving the aim of sustainable green chemistry [30–32].

Contrasting UAE with MWAD procedures, the last uses closed vessels decomposing organic matrices at elevated temperatures and/or pressure, evading analytical volatilization or sample loss. Meanwhile, UAE is an open system, resulting in the temperature and pressure necessary for complete digestion not being competently attained, seriously prejudiced by the sample particle size [30]. Thus, samples were milled until reaching a particle size distribution of fewer than 200 *μ*m.

To assess whether the proposed sample preparation method (UAE) is equivalent to conventional MWAD, five sorghum samples were randomly selected. Digestions were performed in triplicate as indicated in Sections 2.4 and 2.5. The procedure was validated previously by Zaldarriaga Heredia et al. [26], and the performance is shown to be statistically equivalent to the standard AOAC method 985.35 [33].

Ultrasound extraction efficiency is increased as a result of increased medium temperature and increasing sonication time, forming a larger number of cavitation nuclei. Also, the acid type and its concentration affect the UAE. The oxidant combination between HNO_3_ and H_2_O_2_ has been widely used based on the reagents and matrix interaction. The strong oxidizing acid, HNO_3_, is used to enhance organic material mineralization, improving sample digestion via element soluble complex formation [30]. Also, when heating, H_2_O_2_ dissociates to hydroxyl radical (OH) attacking proteins, carbohydrates, and fatty acids present in organic samples [34].

Nowadays, instead of searching for greening chemistry sample preparation procedures, one interesting alternative is the use of diluted reagents. The reduction in amounts of acid for organic samples' digestion leads to relevant advantages such as smaller residual acidity and residue amounts, cost reduction, and lower analytical blanks. In this sense, several recent applications demonstrated the potential of diluted nitric acid solutions [35–39].

Sorghum samples were digested using diluted acid by UAE, and results were compared with MWAD. Multielemental determination was carried out by MIP OES.  Table 2 shows the Cu, K, Mg, Mn, P, and Zn concentrations, with sorghum samples treated by UAE and MWAD. Both methods were statistically compared using a *t*-test (*α* = 0.05; *n* = 3), and there were no significant differences between treatments. In order to compare the precision between MWAD and UAE, an *F*-test was performed, calculating each *F*-value for each element and sample, obtaining a range which was compared with *F*_crit_ = 39.00. In all cases, the precision achieved by UAE was not significantly different from the MWAD method.

According to sustainable and green analytical procedures, UAE could be proposed as an attractive alternative to MWAD, being an interesting, quick, and nonexpensive option for sorghum grains sample preparation. As an added bonus, the UAE time consumption involved in sample pretreatment allows for a larger amount of sample to be run at one time; 50 samples can be digested in the water and ultrasonic bath in 90 minutes, unlike MWAD which has a rotor of 16 positions and can digest 16 samples in 30 min. However, the time required for the MWAD, the cooling of the reactors before the opening, in addition to the washing that is approximately 30 minutes, increases the entire MWAD process time to approximately 90 minutes. The UAE advantage is not only in sample throughput but also the low cost and energy requirements of the US equipment compared to the MW digestor and the simpler availability of the polypropylene metal-free flasks tubes compared to the PTFE tubes. Thus, UAE has the benefit of simplicity of the procedure and speed of determination, improving productivity, with the possibility of being implemented in routine analysis in an analytical laboratory, being in compliance with green and equitable analytical chemistry.

**Figure 1:**
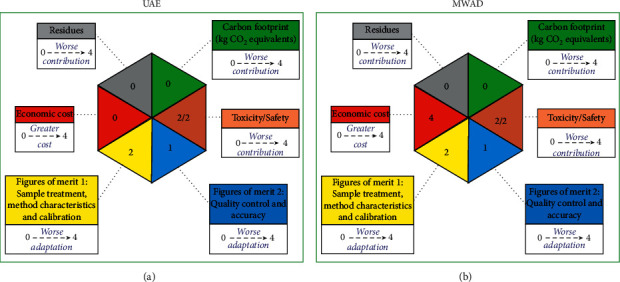
Comparison of Green Analytical Procedure Index for (a) UAE and (b) MWAD sample preparation.

To further highlight these advantages, the greenness of both sample preparation methods was compared by the hexagon tool [40]. This evaluation tool was carried out to assess analytical features of figures of merit, associated chemical and health risks, environmental friendliness, sustainability, and economic cost. The results are depicted in Figures 1(a) and 1(b). As shown in Figure 1, UAE and MWAD offer similar analytical performance; however, the economic cost is higher for MWAD; thus, UAE could be a greener interesting alternative.

### 3.3. Analytical Performance

Afterward verifying the analytical results normality, the Levene test was accomplished to assess the homogeneity of the variance, indicative of the data homoscedasticity. For the linearity evaluation, the goodness of fit was established contrasting the fit lack variance with the pure error variance. Each model suitability was appraised by application of an *F*-test. Better than 0.99 *R*^2^ coefficients were attained for each analyte calibration curve.

The LOD and LOQ of each analyte were calculated, according to the up-to-date International Union of Pure and Applied Chemistry (IUPAC) recommendations, as 3.3 and 10 times the standard deviation (SD) of the measurement blanks (*n* = 10), respectively. Table 1 displays the figures of merit for Cu, K, Mg, Mn, P, and Zn determination by MIP OES after treating samples by UAE. The recommended procedure reaches LOD between 0.6 mg kg^−1^ and 52 mg kg^−1^ and LOQ between 1.9 mg kg^−1^ and 172 mg kg^−1^ for Cu and P, respectively.

For method validation, precision expressed as RSD% obtained using the calibration curves was between 0.27% for Mg to 7.72% for P, reaching good precision and attaining the conventional acceptance criterion (Table 1). A CRM with a similar sample matrix was assessed in order to evaluate the trueness of the proposed UAE procedure under conditions selected in Section 2.4. Data comparison obtained after the application of the developed method with reference values is presented in Table 3. Recoveries between 82% and 110% were observed for all studied analytes. No significant differences were observed for Cu, K, Mg, Mn, P, and Zn. Analytical UAE method precision for the CRM was determined by the repeatability of the measurement set and was less than 6.33%.

The analytical performance of the developed UAE method is suitable for the determination of Cu, K, Mg, Mn, P, and Zn in sorghum flour samples by MIP OES.

### 3.4. Analytical Application

The selected analytical conditions were applied for Cu, K, Mg, Mn, P, and Zn determination in twenty sorghum types, mainly graniferous sorghum, dual-purpose sorghum, and forage sorghum.  Table 4 exposes the concentrations of the 6 analytes found in the 20 sorghum hybrids examined. The concentration of the analyzed element (Cu, K, Mg, Mn, P, and Zn) was similar to previous studies [41–45]. The content of macroelements was in the following order K > P > Mg, while for microelements, Mn and Zn were similar but higher than Cu.

Sorghum is a valuable grain, particularly due to its beneficial health components and its status as a practicable option for people with celiac disease and gluten intolerance. Hybrids with elevated levels of mineral elements such as Mn, Mg, and Zn could be an effective component of functional foods and improve food nutritional quality.

This study reveals that K and P were recorded as dominant minerals in sorghum followed by Mg. The K content was the most variable among hybrids (3641 mg kg^−1^–4806 mg kg^−1^). The P concentrations ranged from 1217 mg kg^−1^ to 1545 mg kg^−1^, and some results were settled below other reports [41, 42]. In both cases, this could be related to the differences between hybrids and the soil type [45]. The Mg content in all hybrids was similar (1218 mg kg^−1^–1525 mg kg^−1^) to wheat flour (970 mg kg^−1^) and higher than cornflour (470 mg kg^−1^) as reported by Danish Food Composition Databank [46]. Since sorghum has a high Mg content, it could be considered a good source of this element.

Regarding microelements concentrations, Mn, Zn, and Cu contents ranged from 15.1 mg kg^−1^ to 25.9 mg kg^−1^, 14.5 mg kg^−1^ to 22.1 mg kg^−1^, and 2.57 mg kg^−1^ to 3.7 mg kg^−1^, respectively. Zinc is known as being a good reducing agent as it can form a complex with sulfates, carbonates, sulfates, phosphates, phytates, and oxalates. Furthermore, Zn is an indispensable cofactor of more than 70 enzymes [47] and is an essential microelement in human nutrition. Its deficiency can trigger growth retardation, dermatitis, recurrent infections, diarrhea, and mental disturbances [48]. A highlight finding in this study is that the Zn content in all hybrids was similar to those reported in whole wheat flour (∼22 mg kg^−1^) and higher than in cornflour (5 mg kg^−1^) [46].

### 3.5. Correlation among Mineral Content

**Figure 2:**
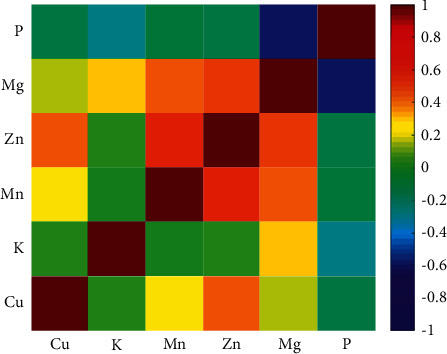
Correlation matrix among variables for the different sorghum genotypes evaluated.

Figure 2 contains a colored representation of the matrix formed by the Pearson correlation coefficients calculated from the mineral concentration of studied sorghum samples. This initial correlation analysis showed the magnitude of the six metal concentrations and their correlation with one another. In general, all variables exhibited low positive or negative correlations with other variables. Highlighting, variable P is slightly negatively correlated with Mg (*r* = −0.62). Other variables negatively correlated were P and K (*r* = −0.30). Significant positive correlations were observed between Mn and Zn (*r* = 0.58), Mn and Zn (*r* = 0.50), Mg and Mn (*r* = 0.42), and Cu and Zn (*r* = 0.42).

However, these correlation coefficients cannot reveal the fact that each group of sorghum hybrids may have a specific behavior. In order to provide a more comprehensive interpretation and to formulate general conclusions, multivariate data analysis was conducted.

### 3.6. Exploratory Analysis by PCA

**Figure 3:**
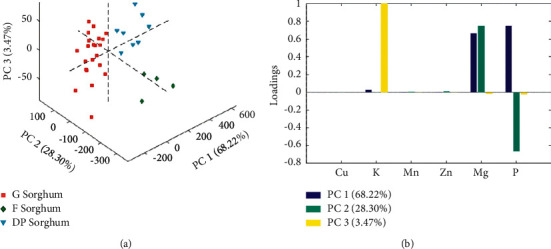
(a) Score scatter plots of the first 3 PCs obtained from PCA applied to a dataset corresponding to each mineral content on sorghum samples. The samples are shown according to the hybrids: G sorghum samples (red square), F sorghum samples (green diamond), and DP sorghum samples (light-blue triangle). Explained variance values of PC1, PC2, and PC3 are displayed in the corresponding axis. (b) Loading bar plots of the first 3 PCs obtained from PCA for each variable.

The elemental composition is valuable to perform multivariate analysis because it can contain information about varieties, geographical origin, or other relevant information from sorghum. In this sense, PCA is a meaningful multivariate tool to observe the behavior of data according to some particularities of samples. Previously, different preprocessing methods were evaluated, and the best one in terms of the explained variance obtained in the models was autoscaling. After finding that some studied variables showed variability on the acquired data, an independent PCA was conducted to assess differences among studied sorghum varieties. As a result of each analysis, the first three PCs were selected to represent data variability.  Figure 3(a) represents the score plots of each dataset defined by the first three principal components calculated by PCA. The percentage of explained variance for individual components is shown on each axis summarizing a total of 99.99%. It can be observed a clear differentiation of three classes related to the three varieties: graniferous sorghum (G), dual-purpose sorghum (DP), and forage sorghum (F). As can be seen in Figure 3(b), this discrimination is related to positive values of PC1 loadings for Mg and P, which permitted to mainly differentiate the G and DP sorghum groups, as well as positive and negative values related to PC2 loadings for Mg and P, respectively, that contribute to discriminate F sorghum of the rest of samples. This result is closely matching with that obtained from the correlation analysis. Finally, PC3 loadings presented high positive values of K that also contributed to integral differentiation among samples.

From the point of view of varieties, this analysis allowed to evidence differences among them in terms of elemental composition and to achieve conclusions about those varieties more relevant from the point of view of human nutrition.

## 4. Conclusion

Regarding green and sustainable analytical procedures, using an ordinary ultrasound bath system for UAE makes sample preparation fast, simple, accurate, and less expensive than MWAD. Sorghum flour samples were suitably mineralized by UAE, enabling their introduction to the MIP OES, not evidencing significant differences with MWAD.

Aiming to explore the nutritional potential of sorghum, MIP OES was used for multielemental determination of sorghum flours in order to assess its mineral profile. Beholding it from food safety control and quality assurance, the proposed method is a magnificent substitute that could be straightforwardly applied in routine analysis laboratories, mainly due to the multielemental capabilities of MIP OES, with the benefit that the UAE is unassuming and respectable with GEAC, thus accelerating analysis times and improving performance and productivity.

The principal component analysis showed different groupings related to the variation in the elemental concentration of the various hybrids. The differentiation between varieties makes it possible to select those with the best nutritional profile for food product formulation.

## Figures and Tables

**Table 1:** Instrumental parameters and analytical figures of merits of the proposed UAE method by MIP OES.

Instrumental parameters	Operational conditions					
Plasma power (W)	1000					
Stabilization time (s)	10					
Background correction	Auto					
Integration time (s)	3					
Nebulizer	OneNeb®					
Spray chamber	Double pass cyclonic					
Sample floe rate (rpm)	15					
Replicates	3					

Analyte	Cu	K	Mg	Mn	P	Zn
Wavelength (nm)	324.754	766.491	285.213	403.076	213.618	213.857
Viewing position	0	−10	−10	0	−10	−10
Nebulizer gas pressure (kPa)	180	220	200	240	220	160
LOD (mg kg ^−1^)	0.6	3.1	11.5	2.0	52	2.0
LOQ (mg kg ^−1^)	1.9	9.5	34.9	6.0	172	6.0
RSD (%)	5.43	4.80	0.27	7.11	7.72	1.48
*R* ^2^	0.99	0.99	0.99	0.99	0.99	0.99

**Table 2:** Analyte concentration determined after the proposed sample preparation method (UAE) compared to the conventional (MWAD) (*n* = 3).

Sample	Cu						K						Mg						Mn						*P*						Zn					
	(mg kg^−1^)						(mg kg^−1^)						(mg kg^−1^)						(mg kg^−1^)						(mg kg^−1^)						(mg kg^−1^)					
	MWAD			UAE			MWAD			UAE			MWAD			UAE			MWAD			UAE			MWAD			UAE			MWAD			UAE		
A	5.5a	±	0.7	3.8a	±	1.1	4539a	±	120	4572a	±	51	1325a	±	125	1502a	±	90	21.2a	±	2.6	15.1a	±	4.0	1333a	±	80	1492a	±	90	18.8a	±	0.4	18.1a	±	0.4
B	3.3a	±	0.2	2.7a	±	0.8	4054a	±	118	4085a	±	150	1245a	±	94	1389a	±	151	24.0a	±	2.7	19.4a	±	2.1	1238a	±	11	1469b	±	56	17.9a	±	1.1	17.9a	±	1.7
C	2.8a	±	0.1	2.5a	±	0.3	4201a	±	186	4232a	±	279	1246a	±	89	1364a	±	242	20.6a	±	0.7	18.4a	±	2.0	1389a	±	218	1289a	±	80	17.0a	±	0.2	16.9a	±	1.2
D	3.1a	±	0.1	3.2a	±	0.3	4218a	±	177	4249a	±	266	1220a	±	26	1287a	±	128	20.3a	±	0.5	22.6a	±	2.9	1185a	±	131	1333a	±	61	15.5a	±	0.5	16.0a	±	1.4
E	3.2a	±	0.1	2.9a	±	0.4	4204a	±	246	4236a	±	369	1334a	±	77	1480a	±	83	21.0a	±	2.2	20.6a	±	2.5	1242a	±	253	1493a	±	92	18.9a	±	1.6	19.5a	±	0.7

Values are expressed as mean (n = 3). If the letters between treatments (MWAD and UAE), for each analyte and for each sample, are not equal, they mean that there is a statistically significant difference (*p* < 0.05). MWAD: microwave-assisted digestion; UAE: ultrasound-assisted extraction.

**Table 3:** Analyte concentration certified in reference material and determined concentrations in certified reference material (*n* = 3).

Analyte	Certified values (mg kg^−1^)			Determined values (mg kg^−1^)			Recovery %
Cu	0.66	±	0.08	0.67	±	0.04	101
K	1290	±	70	1249	±	3	97
Mg	180	±	20	160	±	6	89
Mn	1.55	±	0.08	1.52	±	0.29	98
P	610	±	30	668	±	102	110
Zn	2.90	±	0.30	2.37	±	0.17	82

Reference certified material: Maize Flour (NCS ZC 73010 Mealie).

**Table 4:** Mean analyte concentrations (*n* = 3) and standard deviations found in sorghum flours.

Sample	Analyte																		Sorghum commercial types	Hybrid
	Cu (mg kg^−1^)			K (mg kg^−1^)			Mg (mg kg^−1^)			Mn (mg kg^−1^)			P (mg kg^−1^)			Zn (mg kg^−1^)				
I	2.8	±	0.3	3641	±	59	1255	±	35	21.6	±	0.1	1394	±	25	16.7	±	3.4	Graniferous sorghum	TOB 41 T
II	3.7	±	0.6	4060	±	295	1328	±	49	20.8	±	0.6	1315	±	50	19.5	±	3.5	Graniferous sorghum	TOB EXP 903
III	3.8	±	1.1	4572	±	51	1502	±	90	15.1	±	4.0	1492	±	90	18.1	±	0.4	Graniferous sorghum	ACA 548
IV	2.6	±	0.1	4375	±	338	1218	±	63	17.7	±	0.4	1482	±	65	14.5	±	3.3	Graniferous sorghum	ACA XP GR 209
V	2.9	±	0.2	4329	±	431	1319	±	68	25.9	±	0.3	1276	±	51	16.8	±	2.9	Graniferous sorghum	ACA GR 233
VI	2.7	±	0.8	4085	±	150	1389	±	151	19.4	±	2.1	1469	±	56	17.9	±	1.7	Graniferous sorghum	ARGENSOR 110 T
VII	3.2	±	0.4	4528	±	44	1329	±	28	19.7	±	2.6	1459	±	50	19.8	±	1.6	Dual-purpose sorghum	ARGENSOR 151 DP
VIII	3.4	±	0.8	4256	±	181	1406	±	89	22.6	±	2.1	1217	±	43	22.1	±	0.9	Graniferous sorghum	GEN 11T
IX	3.1	±	0.3	3917	±	929	1356	±	84	21.6	±	0.9	1397	±	29	18.4	±	0.2	Graniferous sorghum	GEN 311 T
X	2.5	±	0.3	4232	±	279	1364	±	242	18.4	±	2.0	1289	±	80	16.9	±	1.2	Dual-purpose sorghum	GEN 417 SLT
XI	3.2	±	0.3	4249	±	266	1287	±	128	22.6	±	2.9	1333	±	61	16.0	±	1.4	Graniferous sorghum	SUMMER II
XII	2.9	±	0.4	4236	±	369	1480	±	83	20.6	±	2.5	1493	±	92	19.5	±	0.7	Graniferous sorghum	SPRING T 60
XIII	3.3	±	0.2	4269	±	40	1525	±	93	23.6	±	0.0	1336	±	10	21.8	±	0.1	Graniferous sorghum	EXP 483
XIV	3.2	±	0.2	3822	±	58	1285	±	36	24.8	±	1.7	1457	±	21	18.6	±	2.5	Graniferous sorghum	TAMAR 2 GR
XV	2.9	±	0.4	4401	±	456	1370	±	40	21.5	±	2.0	1473	±	60	18.3	±	0.3	Graniferous sorghum	HS 26 LT
XVI	2.9	±	0.2	4806	±	975	1411	±	49	20.2	±	1.1	1490	±	31	15.4	±	6.8	Graniferous sorghum	PS 55
XVII	3.0	±	0.4	4379	±	138	1367	±	34	21.2	±	1.8	1506	±	14	18.5	±	0.3	Forage sorghum	GS1
XVIII	2.8	±	0.2	4382	±	511	1410	±	82	20.0	±	0.2	1449	±	10	17.7	±	0.8	Forage sorghum	GS2
XIX	3.1	±	0.1	4380	±	379	1248	±	23	23.8	±	1.7	1545	±	50	18.3	±	0.5	Forage sorghum	GSD 3
XX	2.9	±	0.6	4749	±	194	1465	±	76	19.3	±	4.1	1482	±	20	18.1	±	3.9	Forage sorghum	GSD 4

## Data Availability

Data used to support the findings of this study are available on request to the corresponding author (marianelasavio@gmail.com).
